# Spiritual Evidence

**Published:** 1891-08

**Authors:** 


### SPIRITUAL EVIDENCE.

While our spiritual senses are closed we have no sensible recognition of the spirits and spiritual things around us, and therefore we ought to accept other reliable evidence of their existence, as do those who are born blind and deaf accept various evidences of the existence of light and sound. We do not accept the modern theory of the motions and relations to each other of the heavenly bodies because Copernicus declared it and astonomers since him believe it to be correct ; but we are convinced of its correctness by its explanations of our seasons, the phases of the moon, eclipses and the possibility of their prediction, the motion of moons around some of the planets, and from many other things. So are thousands of people now convinced of the truth of what Swedenborg writes concerning the spiritual world and the formation and government of nature from it, as well as of his explanation of the spiritual or internal sense of the Word of God, and the truth of the doctrine of correspondence of natural with spiritual things, not because he says he saw, heard and perceived the things he mentions, but by the many proofs that these things must be so, fully comprehended by themselves.—*Mount Joy Herald.*

